# Totipotency in the mouse

**DOI:** 10.1007/s00109-017-1509-5

**Published:** 2017-01-19

**Authors:** Guangming Wu, Lei Lei, Hans R. Schöler

**Affiliations:** 1 Department of Cell and Developmental Biology, Max Planck Institute for Molecular Biomedicine, Röntgenstrasse 20, 48149 Münster, Germany; 2 Department of Histology and Embryology, Harbin Medical University, 194 Xuefu Road, Nangang District, Harbin, 150081 China; 3 Medical Faculty, University of Münster, Domagkstr. 3, 48149 Münster, Germany

**Keywords:** Totipotency, Zygote, Transcription factors

## Abstract

In mammals, the unicellular zygote starts the process of embryogenesis and differentiates into all types of somatic cells, including both fetal and extraembryonic lineages—in a highly organized manner to eventually give rise to an entire multicellular organism comprising more than 200 different tissue types. This feature is referred to as totipotency. Upon fertilization, oocyte maternal factors epigenetically reprogram the genomes of the terminally differentiated oocyte and spermatozoon and turn the zygote into a totipotent cell. Today, we still do not fully understand the molecular properties of totipotency. In this review, we discuss recent findings on the molecular signature and mechanism of transcriptional regulation networks in the totipotent mouse embryo.

## Introduction

**Fig. 1:**
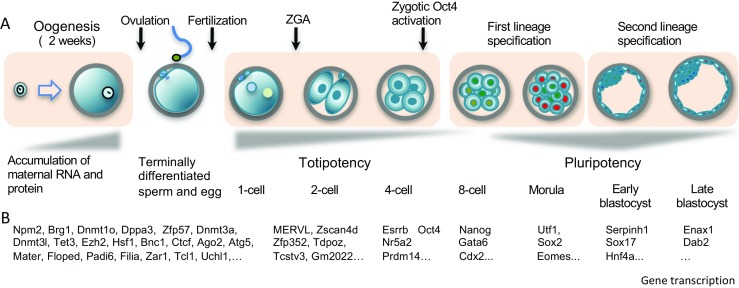
Mouse preimplantation development. **a** Mature oocytes are ovulated from the ovary into the oviduct and fertilized by sperm to establish totipotent zygotes that divide and become blastocysts, and finally implant in the uterus at embryonic day 4.5. **b** After fertilization, stored maternal factors trigger zygotic genome activation (ZGA) that results in formation of a totipotent zygote with a unique two-cell-specific gene-expression profile, followed by waves of transcription activations of lineage specific genes during preimplantation development

Multicellular organisms typically originate from a single totipotent cell, the zygote. In plants, structurally and functionally specialized cells of leaves, roots, stem, floral parts, and endosperm retain the potential to revert back to the undifferentiated state and form entire new plants, irrespective of their ploidy level (haploid, diploid, or triploid). The potential of terminally differentiated cells to regenerate whole plants was referred to as “cellular totipotency” by the remarkable German plant physiologist Göttlieb Haberlandt in his famous address to the German Academy in 1902 [1]. Now, regeneration of totipotency from isolated single plant cells is well demonstrated [2]. However, in the mouse, totipotency seems to be restricted up to two-cell embryos. The term “Totipotency” is defined by two related but different criteria: (1) the ability of a single cell to contribute to all cell lineages, including the TE, of an organism; and (2) more stringently, the ability of a single cell to develop into a complete organism [3, 4]. The zygote is the ultimate totipotent cell (Fig. 1). Blastomeres from two-cell–stage embryos also fulfill the more stringent definition for totipotency [5–7]. Prior to the first lineage segregation, totipotency is lost gradually [8]. Some blastomeres from eight-cell–stage embryos contribute to the development of all lineages in chimeric mice [9–11], and thus provide evidence for totipotency based on the less stringent definition. In this review, we discuss present-day understanding of the transcription factor networks and epigenetic reprogramming involved in the emergence of totipotency in the mammalian embryo.

## Establishment of totipotency

### Zygotic genome activation

Following fertilization, maternal factors play a leading role in epigenetically resetting the parental DNA and histones across the genome of the zygote, thereby preparing for whole-genome activation and establishment of totipotency. A burst of transcription—known as zygotic genome activation (ZGA)—begins at the late one-cell stage and peaks at the two-cell stage in the mouse [12]. ZGA is characterized by more efficient use of TATA-less promoters [13]; activation of repetitive elements [14], particularly endogenous retrotransposons, e.g., murine endogenous retrovirus with a leucine tRNA primer binding site (MERVL) at the two-cell stage as a marker for totipotent cells [15]; uncoupling of transcription and translation in zygotes [16]; and activation of enhancers for transcription in two-cell embryos [17]. ZGA provides the first step in the establishment of totipotency.

### Maternal factor storage

During oogenesis, the volume of oocytes dramatically increases to accommodate the storage of maternal factors (RNA, proteins) required for establishing totipotency and ZGA, such as nucleoplasmin (NPM) 2 [18], and the subcortical maternal complex (SCMC, including Mater, Tle6, Floped, Padi6, Filia) [19]. In the growing oocytes, subcortical ribonucleoprotein (RNP) particle domains (SCRDs) are formed to serve as the storage compartment of maternal messenger RNA (mRNA) [20]. Maternally accumulated yes-associated protein (YAP) has recently been identified to play a critical role in ZGA [21]. However, the paucity of biological materials from mouse oocytes and zygotes has hampered our effort to understand how maternal factors reprogram cells to totipotency [22]. Further identification of key maternal regulators and their functions could greatly facilitate studies for improving chromatin reprogramming [23, 24].

### Histone modifications

Hyperaccessibility of chromatin by transcriptional machinery is a prerequisite for ZGA. Chromatin accessibility is largely determined by histone modifications of its N-terminal tails (“marks”), which acts as a fundamental epigenetic regulator to control the gene expression during embryo development in mammals.

There are two major types of histone modifications involved in regulation of gene expression during the ZGA: lysine acetylation and lysine (tri)methylation. H4 acetylation makes pronucleus permissive for active transcription [25]. Loss of the maternal Brg1, a component of the ATP-dependent chromatin remodeling SWI/SNF complex, results in reduced levels of 30% of zygotic genes and arrest at two-cell, demonstrating that chromatin remodelers that induce to acetylation are required for mouse embryogenesis [26].

The opposing marks histone H3 lysine 4 trimethylation (H3K4me3) and histone H3 lysine 27 trimethylation (H3K27me3) at gene promoter regions are associations with active and repressed genes, respectively. Following fertilization, H3K4me3 and H4 acetylation in the paternal genome are responsible for a minor ZGA. They are depleted in late zygotes stage but reestablished on promoter regions during the major ZGA at the late two-cell stage [27, 28]. On the maternal genome, a noncanonical (nc) form of H3K4me3 (ncH3K4me3) is present broadly in oocytes and zygote and overlaps almost exclusively with partially methylated DNA domains. The ncH3K4me3 is erased in the late two-cell embryos [27]. Active removal of broad H3K4me3 domains by the lysine demethylases KDM5A and KDM5B is required for ZGA and is essential for early embryo development [29].

### Protamine-to-histone replacement

At the time of fertilization, the chromatin molecules of the paternal and maternal genomes exhibit different epigenetic marks and organization. The paternal genome is haploid, and most of it is packaged densely, with protamines rather than histones, while the maternal genome is diploid, as it arrests at metaphase II, and is packaged with histones. After a sperm penetrates the cytoplasm of the oocyte, the paternal genome decondenses, enabling protamine removal and repackaging with the stored maternal histones in the absence of DNA replication, while the maternal genome completes meiosis. These newly integrated histones possess a transcriptionally permissive pattern of modifications, including H4 hyperacetylation [25] and H3K9 and H3K27 monomethylation [30]. Of note, when round spermatids, which contain DNA that is still associated with histones, are injected into oocytes by round spermatid injection (ROSI), paternal genome failed to undergo active DNA demethylation, but when mature sperm, which contain DNA associated mainly with protamines, are injected into oocytes by intracytoplasmic sperm injection (ICSI), active paternal genome demethylation is observed [31]. These results indicate that the protamine-histone exchange may cause the pronounced demethylation of the paternal DNA in the zygote. However, as both ROSI- and ICSI-derived embryos have the same likelihood of developing to term, paternal genome demethylation mediated by protamine-histone exchange is not an essential step in ZGA and establishment of totipotency [32].

### Histone variant H3.3

During preimplantation development, striking changes in epigenetic modifications in the form of deposition of histone variants, reestablishment of histone marks, and DNA demethylation occur throughout the genome.

Both canonical histones (H2A, H2B, H3, and H4) and variant histones (which have sequence homology and structural similarity with canonical histones, but harbor specialized functions and play essential roles in chromatin reprogramming) are incorporated into chromatin throughout the first cell cycle of the zygote. Variant histones preferentially are deposited into specific genomic regions to form nucleosomes with unique biophysical characteristics. As one of the three variants of histone H3 in mammals, H3.3 differs from canonical H3 in only four amino acids and incorporates into chromatin in both a replication-independent and a replication-coupled manner. H3.3 interacts with the chaperones HIRA and Daxx/ATRX and is enriched in transcriptionally active regions [24]. H3.3 also localizes to telomeres, where its presence depends upon ATRX [33]. Following fertilization in the mouse, maternal H3.3 is deposited by HIRA onto paternal chromatin during the protamine-to-histone exchange [34], an essential step for oocyte-mediated reprogramming [35]. H3.3 is also required for maintaining chromatin in the decondensed state in early mouse embryos by antagonizing linker H1, an activity dependent on H3.3 lysine 36 [36].

### Active DNA demethylation

The genome-wide cytosine methylation profile differs among cell types, and it functions as a form of memory of the cell’s identity [37]. 5-methylcytosine (5mC) is present mostly in CpG sequences [37–39]. Methylation occurs globally in mammalian genomes at various loci including genes, transposons, repeat sequences, and intergenic DNA [40]. The enzymes that methylate cytosine to form 5mC have been well characterized. DNA methyltransferase (DNMT) 1 preferentially methylates hemi-methylated cytosines in CpG sequences and thus acts as a methyltransferase that maintains genome-wide methylation patterns during replication [41–43]. DNMT3A and DNMT3B can methylate unmethylated CpG sequences and hence function as de novo methyltransferases [44]. DNMT3L has no catalytic activity but recruits DNMT3A and DNMT3B to their target sequences by recognizing nucleosomes that carry unmethylated histone H3 lysine 4 (H3K4) [45–49].

In concordance with histone acquisition, the paternal genome undergoes genome-wide loss of DNA methylation via an active mechanism prior to the start of DNA replication [50, 51].

Recent studies have found a new mechanism of active demethylation involving prior modification of methylated cytosine and nucleotide excision and repair. 5-hydroxy-methylcytosine (5hmC), a stable hydroxylated metabolite of 5mC, was first identified in the genome of T-even bacteriophages [52], and it is produced as an oxidation damage product of 5mC [53, 54]. Subsequent studies have found this to actually be a physiologically relevant DNA modification in mammals, e.g., in mouse neurons and embryonic stem cells (ESCs) [55, 56]. The hydroxylation of 5mC into 5hmC is catalyzed by a family of dioxygenases—the ten-eleven translocation (TET) 1/2/3 proteins. TET proteins convert 5mC into 5hmC [56], and further into 5-formylcytosine (5fC) and 5-carboxymethylcytosine (5caC) for excision [57, 58]. As 5hmC has a significantly lower affinity for methyl-CpG binding proteins [59], it may be directly involved in epigenetic regulation. Indeed, genome-wide DNA demethylation in the zygote is accompanied by Tet3-driven genome-wide oxidation of 5mC into 5hmC [60–62]. Such 5hmC formation does not account for the initial loss of paternal 5mC in the early pronuclear stage, but it is dependent on the activity of zygotic Dnmt3a and Dnmt1, suggesting that Tet3 is targeting de novo methylated sites for the accumulation of 5hmC [63].

Although recent sequence data has shown active demethylation in maternal DNA as well [64, 65], high levels of 5hmC are detected only in the paternal genome of the zygote [60, 66]. A maternal knockout of *Tet3* has been shown to prevent both elevation of 5hmC and reduction of 5mC levels in the paternal genome, impair promoter demethylation of *Oct4* (*Pou5f1*) and *Nanog*, delay the activation of a paternally derived *Oct4* transgene, and cause frequent death of the resulting embryos [62]. These findings suggest that during normal development, TET3 converts 5mC into 5hmC in the paternal genome, and that TET3-mediated hydroxylation of 5mC accounts for at least some of the active DNA demethylation of the paternal genome. The ubiquitin ligase Cullin-ring finger ligase-4 (CRL4) has recently been reported to induce TET3 activity and plays an essential role in female fecundity [67], further strengthening the importance of active DNA demethylation during embryonic development.

### Activation of embryonic Oct4 expression

The maternal octamer-binding transcription factor 4 (Oct4), encoded by the gene Pou5f1 hereafter referred to as Oct4, is at the top of the pluripotency regulatory hierarchy in pluripotent cells [62, 63]. However, several recent studies using conditional genetic depletion of maternal Oct4 have found that the oocytes of *Oct4* ^*flox/flox*^ */ZP3* ^*Cre/+*^ female mice are capable of completing full-term development after fertilization, indicating that Oct4 is not required for initiating totipotency or pluripotency in embryos [68–70]. The two cell-like ESCs are found to lose Oct4 expression at the protein level [15], suggesting that Oct4 activation in early embryos demarcates pluripotency and totipotency. Still, Oct4 is at the top of the pluripotency regulatory hierarchy in pluripotent cells [71, 72]. It forms a positive feedback loop [73] and is essential for maintaining pluripotency [74]. Therefore, identifying upstream factors of *Oct4* activation in early embryos is critical for understanding the molecular regulation network of totipotency and transition from totipotency to pluripotency. There are a few transcriptional factors found to be involved in the regulation of *Oct4* expression. In proliferating stem cells, Promyelocytic leukemia (Pml) protein, along with the transcription factors TR2, SF1, and Sp1, and the Brg1-dependent chromatin remodeling complex (BRGC), associates with the *Oct4* promoter to maintain a nucleosome-free region for *Oct4* gene expression [75]. Cancer-associated factor Tpt1 has been reported to activate the transcription of *Oct4* and *Nanog* in transplanted somatic nuclei in Xenopus oocytes [76], but knockdown of *Tpt1* by small interfering RNA (siRNA) does not reduce Oct4 expression in mouse embryos [68]. The maternal transcription factor spalt-like transcription factor 4 (Sall4) binds to the *Oct4* distal enhancer (DE), and evidence shows that injection of *Sall4* siRNA into zygotes knocking down *Sall4* mRNA levels by 50% leads to a 70% reduction of *Oct4* expression levels, suggesting that Sall4 is a transcriptional activator of *Oct4* expression [77]. Contradictorily, knockdown of *Sall4* by injection of more efficient *Sall4* siRNA into maternal *Oct4*-deficient zygotes—to avoid any possible effect of maternal Oct4 as a positive autoregulator—does not lead to any *Oct4* expression changes at the blastocyst stage [68]. The nuclear receptor subfamily 5, group A, member 2 (Nr5a2), also known as liver receptor homolog-1 (LRH-1), was found to maintain Oct4 expression at the epiblast stage of embryonic development, by binding to the proximal enhancer (PE) and proximal promoter (PP) regions of *Oct4*, but to play no evident role in the self-renewal of ESCs [78]. However, Nr5a2 can induce epiblast stem cells into ground-state pluripotency—a basal proliferative state that is free of epigenetic restriction [79], and to replace Oct4 in the reprogramming of somatic cells into pluripotent cells [80]. As a component of an active DNA demethylase, activation-induced cytidine deaminase (*AID*) has also been shown to be required for Oct4 activation during reprogramming [81]. A genome-scale RNA interference (RNAi) screen in ESCs has identified components of the Pol II-associated factor 1 (Paf1) complex that have strong effects on *Oct4* expression, and shown that Paf1C overexpression blocks the differentiation of ESCs while Paf1C knockdown causes expression changes similar to those caused by Oct4 or Nanog depletion [82]. Studies in search for oocyte master genes have revealed a novel oocyte-specific eukaryotic translation initiation factor 4E (*Eif4eloo*) [83] and a large number of oocyte-specific genes with yet unknown functions, such as those belonging to the homeodomain transcription factor Obox family [84]. To this day, it is unclear how *Oct4* expression is activated in the embryo.

## Molecular signature of totipotency

Unlike the case for pluripotency, the mechanism underlying the molecular regulation of totipotency remains largely unknown. In mice, only the zygote and two-cell-stage blastomeres can generate an entire organism on their own, and are therefore regarded as totipotent cells [6]. The morphology of two-cell embryos is characterized by lack of 4,6-diamidino-2-phenylindole (DAPI)–stained chromocenters in the nucleus [85], and the high chromatin mobility at the two-cell stage progressively decreases with development [86]. The transcriptional profile of two-cell embryos is characterized by activation of major satellites, MERVL, and two-cell-specific genes, such as Eif1a-like genes (which include Gm5662, Gm2022, Gm4027, BB287469, Gm2016, Gm21319, Gm8300, and Gm10264), Zscan4 genes (Zscan4b–Zscan4f), Zfp352, and Tdpoz genes (Tdpoz1–Tdpoz5) [87]. A recent study has demonstrated that depletion of either the p150 or p60 subunit of chromatin assembly factor-1 (CAF-1) in ESCs leads to the formation of 2-cell-like cells with a morphology and transcriptional profile similar to those of two-cell-stage embryos [88]. As CAF-1 performs the first step of the chromatin assembly process by bringing H3 and H4 in close proximity to the daughter DNA strands [89] and as the absence of functional CAF-1 delays nucleosome assembly [90], the inefficiency of chromatin assembly in two-cell embryos has been proposed to be a key mechanism in establishing totipotency [88].

Retrotransposon transcripts contribute a significant portion to the transcriptome during ZGA. Retrotransposons can also act as alternative promoters in the activation of protein-coding genes by generating chimeric transcripts with retrotransposon gene junctions [14]. The most active LINE-1 retrotransposons form a stimulatory auto-enhancing loop, indicating that maternal retrotransposon transcripts could activate endogenous retrotransposons after fertilization [91].

*Zscan4* is activated during ZGA [92] and can act as an activator of spontaneous telomere sister chromatid exchange (T-SCE) and telomere elongation in mouse ESCs [93]. Knockdown of *Zscan4* by siRNAs delays progression from the two-cell to four-cell stage, and thus leads to the formation of blastocysts that fail to implant or proliferate in blastocyst outgrowth culture [92]. Zscan4 is essential for generation of induced pluripotent stem cells (iPSCs), and its ectopic expression can activate early embryonic genes and improve the efficiency of iPSC generation [94]. Expression of the Zscan4 gene family plays important roles in genome stability and maintenance of telomeres [93]. Of note, the absence of nuclear receptor subfamily 0, group B, member 1 (Nr0b1), also known as Dax1, which is an important component of the transcription factor network that governs pluripotency in mouse ESCs, also leads to the overexpression of two-cell embryo-specific transcripts, including Zscan4c, preventing normal self-renewal by inducing arrest at the G2 phase followed by cell death [95].

Furthermore, another recent study has described a small transient ESC/iPSC population with fluctuating expression of a particular retrotransposon, MERVL and a transcriptome that closely resembles that observed in the blastomeres of the totipotent, two-cell embryos [15], indicating that some features of totipotent cells can be regained occasionally in pluripotent cells. This phenomenon provides us with a novel way of studying certain aspects of totipotency. However, the study did not prove these two-cell like cells to be totipotent according to the stringent criteria, in which a single totipotent cell can develop into a complete organism. Moreover, near-complete (95%–99%) knockdown of muERV-L transcripts by three different siRNA duplexes did not interfere with full-term embryonic development (unpublished data), suggesting that the transcripts of retrotransposon elements are not involved in the regulation of totipotency, but rather occur as a consequence of global DNA demethylation prior to ZGA.

Recent progress in identifying two-cell marker genes and new players in the genome-wide demethylation process has shed light on the molecular mechanism governing totipotency. Nevertheless, many questions still remain unanswered. To date, no totipotent cell lines have been established in vitro. The upstream “master” signals that trigger the establishment of totipotency have not yet been identified. Given the distinct phases of ZGA in the mouse, it is likely that multiple regulators/cofactors are associated with regulators of ZGA to ensure temporal gene activation for establishing totipotency. It would be interesting to see how the upstream signals relate to histone replacement or modification, genome-wide DNA demethylation, ZGA, and the expression of two-cell-specific genes, particularly those of maternal origin. The identification of two-cell marker genes has moved us closer to solving the fundamental question in developmental biology of how totipotency is established.
